# Sub-satisfactory recanalization of severe middle cerebral artery stenoses can significantly improve hemodynamics

**DOI:** 10.3389/fcvm.2022.922616

**Published:** 2022-09-29

**Authors:** Kun Zhang, Wei Ren, Tian-Xiao Li, Zi-Liang Wang, Bu-Lang Gao, Jin-Chao Xia, Hui-Li Gao, Yong-Feng Wang, Jian-Jun Gu

**Affiliations:** Department of Intervention, Henan Provincial People's Hospital, Zhengzhou, China

**Keywords:** middle cerebral artery, arterial stenosis, hemodynamics, endovascular recanalization, stent angioplasty

## Abstract

**Purpose:**

To investigate the effect of sub-satisfactory stent recanalization on hemodynamic stresses for severe stenoses of the middle cerebral artery (MCA) M 1 segment.

**Materials and methods:**

Patients with severe stenoses of the MCA M1 segment treated with endovascular stent angioplasty were retrospectively enrolled. Three-dimensional digital subtraction angiography before and after stenting was performed; the computational fluid dynamics (CFD) analysis of hemodynamic stresses at the stenosis and normal segments proximal and distal to the stenoses was analyzed.

**Results:**

Fifty-one patients with severe stenosis at the MCA M1 segment were enrolled, with the stenosis length ranging from 5.1 to 12.8 mm (mean 9 ± 3.3 mm). Stent angioplasty was successful in all (100%) the patients. The angiography immediately after stenting demonstrated a significant (*P* < 0.05) decrease in MCA stenosis after comparison with before stenting (31.4 ±12.5% vs. 87.5 ± 9.6%), with residual stenosis of 15–30% (mean 22.4 ± 3.5%). Before stenting, the total pressure was significantly higher (*P* < 0.0001), while the WSS, velocity, and vorticity were all significantly decreased (*P* < 0.0001) at the normal arterial segment proximal to the stenosis, and the total pressure, WSS, velocity, and vorticity were all significantly decreased (*P* < 0.0001) at the normal arterial segment distal to the stenosis compared with those at the stenosis. After sub-satisfactory stenting recanalization, all the hemodynamic stresses proximal or distal to the stenosis and at the perforator root were improved compared with those before stenting and were similar to those after virtual stenosis removal.

**Conclusion:**

Sub-satisfactory recanalization of severe MCA stenoses can significantly improve the hemodynamic status for cerebral perfusion at the stenoses.

## Introduction

Middle cerebral artery (MCA) atherosclerotic stenosis is an important cause of recurrent cerebral ischemic strokes (1–6), and stent angioplasty is a valuable approach for treatment of severe MCA stenosis, mainly by endovascular revascularization to improve cerebral blood flow perfusion and prevent the occurrence of MCA stenosis-related hypoperfusion cerebral infarction (7, 8). The Enterprise stent (Codman, Raynham, MA) was the second one to receive US FDA approval for stent-assisted coiling and the first one with a closed-cell design. It has now become a well-established tool for treatment of aneurysm and intracranial atherosclerotic stenosis as an off-label application (9–16). The second-generation Enterprise stent is a further development with a changed closed-cell design of the strut geometry to improve its adherence to the vascular wall, especially in curved arteries (10). Clinically, undersized angioplasty and the closed-cell Enterprise stent have been applied to treat severe intracranial atherosclerotic stenoses resistant to dual-antiplatelet medications, resulting in favorable clinical and angiographic outcomes with reduced intraprocedural complications (12). The “sub-satisfactory” revascularization by undersized angioplasty and stenting is able to effectively improve blood flow and cerebral perfusion; however, no studies have been performed to investigate changes in hemodynamic stresses (pressure, wall shear stress or WSS, vorticity, and velocity) in the stenotic artery before and after stent angioplasty. It had been hypothesized that “sub-satisfactory” revascularization in undersized angioplasty and stenting would be sufficient to improve blood flow and cerebral perfusion in terms of hemodynamic status. This study used computational fluid dynamics (CFD) to analyze local hemodynamic changes in MCA stenoses before and after stenting in patients with severe MCA stenoses who underwent balloon angioplasty and Enterperise stent deployment to improve the effect and understanding of “sub-satisfactory” stent angioplasty for treatment of severe stenosis of MCA.

## Materials and methods

### Subjects

This retrospective study was approved by the ethics committee of a tertiary hospital (2018-052), and all the patients had given their signed informed consent to participate. The inclusion criteria were similar to those in the SAMMPRIS trial (17), including asymptomatic and symptomatic patients with non-disabling stroke or transient ischemic attack 30 days from last onset. All patients with severe atherosclerotic stenoses of the MCA M1 segment (≥70%) and Tmax >6 s perfusion deficit confirmed by computed tomography (CT) and magnetic resonance (MR) perfusion-weighted imaging, and those being treated by stent angioplasty and displaying one or more perforators near the stenosis were enrolled. Patients with one or more perforators near the stenosis were enrolled because we would like to detect blood flow improvement in the perforators after the sub-satisfactory recanalization. However, digital subtraction angiography could not detect all patients with suitable perforators for analysis, and only patients with clear perforators shown on three-dimensional imaging were enrolled. The exclusion criteria were patients with bad imaging display of arterial segments distal to the stenosis, non-atherosclerotic (arteritis or induced by other factors, such as Moyamoya disease) intracranial arterial stenosis, tandem extracranial or intracranial stenosis (70–99%) proximal or distal to the target intracranial stenosis, arterial dissection, acute cerebral infarction within 2 weeks, intracranial tumors or hemorrhages, severe dysfunction of the heart, liver, and kidney, and mRS score >3 points. The atherosclerotic stenosis on imaging was evaluated using the criteria defined in the studies of the Warfarin-Aspirin Symptomatic Intracranial Disease (WASID) based measurements (18, 19), with the stenosis between 0–69% defined as mild to moderate stenosis and 70–99% as severe stenosis.

### Stent angioplasty

All the patients underwent endovascular stent angioplasty under general anesthesia. Dual antiplatelet inhibition was performed with aspirin (100 mg/day) and clopidogrel (75 mg/day) 3 days before the procedure, and a weight-based intravenous heparin bolus injection was conducted to achieve an activated clotting time of 200–350 s before guide catheter placement. The Seldinger technique was used to puncture the femoral artery followed by insertion of an 8F arterial sheath in the artery. After a micro-guidewire was used to navigate through the stenosis, a Gateway balloon catheter (Stryker, United States) was placed on the stenosis and inflated slowly to dilate the stenosis. The diameter of the balloon selected for pre-dilatation was 1.5–2.75 mm (mean 1.7 ± 0.2), and the length was 9–20 mm (mean 13.5 ± 2.6), with the pre-dilatation pressure ranging from 2 to 8 atm (mean 5.3 ± 2.4) and the pre-dilatation time ranging from 30 to 75 s (mean 45.7 ± 34.8). Then, a Prowler micro-catheter (Codman, United States) was used to navigate the stenosis for deployment of the Enterprise stent (Codman, United States) according to instructions for use of the manufacturer. The stent selected for deployment was 6–10 mm longer than the stenosis, with 3–5 mm over each end of the stenosis. Angiography was performed to confirm the appropriate deployment of the stent, and the residual stenosis was assessed based on normal distal arterial diameter, and successful stenting was defined as residual stenosis < 30%.

In this study, a Gateway balloon catheter (Stryker, United States) was used. The size of the balloon catheter was selected according to the measurement of MCA stenosis, and the balloon diameter was selected to be no more than 80% of that of normal blood vessels at the distal end of the lesion. Based on studies in the literature (20, 21), the MCA arterial diameter is 2.51 ± 0.2 mm for women and 2.54 ± 0.25 mm for men on MRI imaging (21), and it is similar to that measured on cerebral angiography, which is 3.05 ± 0.39 mm for women and 3.35 ± 0.43 mm for men (20). The range of balloon size in this study was 1.5–2.75 mm, and ~80% of the MCAs had a diameter within this range.

### CFD

**Figure 1:**
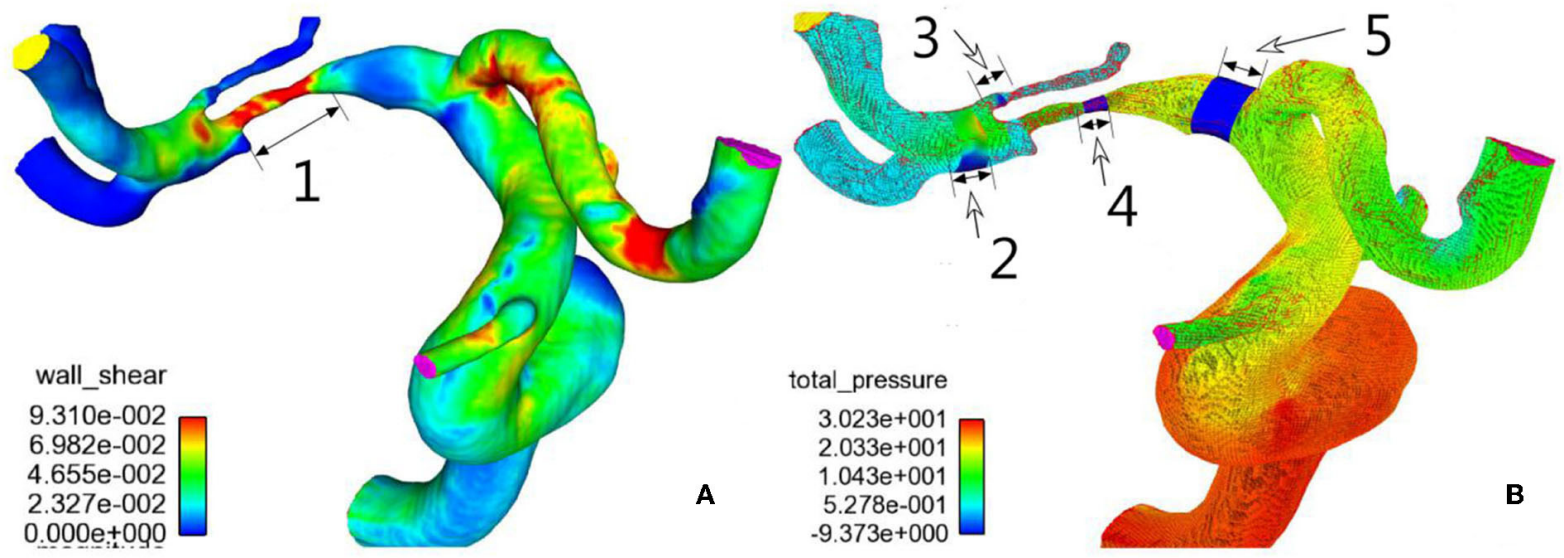
Hemodynamics of M1 stenosis in the middle cerebral artery (MCA). **(A)** Wall shear stress distribution was demonstrated on the cerebral arteries. **(B)** Pressure distribution on cerebral arteries. 1, M1 stenosis; 2, normal artery distal to the stenosis; 3, root of the perforator; 4, stenosis; 5, normal artery proximal to the stenosis. Hemodynamic sampling was performed on the above location.

The three-dimensional (3D) DICOM data of all the patients were collected for analysis, and the Amira software (version 4.1.2, Visage Imaging, San Diego, CA, United States) was used for visualization and removal of small or unnecessary arteries. Surface smoothing of the 3D vascular datasets was conducted with the MeshLab software (MeshLab versions 1.2.3, Visual Computing Lab; *Istituto di Scienza e Tecnologie dell'Informazione, Consiglio Nazionale delle Ricerche*). Virtual removal of the arterial stenosis and virtual repair of the artery were performed with the Meshlab software. Sharc Harpoon V4.3a (http://www.sharc.co.uk/html/downloads.htm) was used for generation of high-resolution 3D polyhedral mesh at a count of 1,000,000 cells. Finite-volume solution was performed with the Ansys 12.0.16 software (Ansys, Lebanon, NH, United States) using the laminar double-precision steady regimen, which assumed blood viscosity of 0.004 Pa S, rigid nonslip wall conditions, and density of 1,050 kg/m^3^ (22). The inlet velocity was set at 0.36 m/s, and the outlet pressure was set at 0 Pa. The whole cardiac cycle was simulated for 0.8 s and used for calculation of mean values of three cardiac cycles. The Ensight software package (Version 9; CEI, Apex, NC, United States) was used for postprocessing analysis of pressure, WSS, vorticity, and velocity proximal and distal to the stenosis, at the stenosis, and at the root of the perforating arterial branch before and after stenting and after virtual removal of the stenoses (Figure 1).

### Follow-up

Follow-up was conducted on all the patients for angiographic evaluation of in-stent restenosis 3–12 months after the stenting and possible ischemic stroke related to the stenting.

### Statistical analysis

All the data were analyzed with the SPSS 17.0 software (IBM, Chicago, IL, United States). Continuous measurement data in normal distribution were expressed as mean ± standard deviation and tested by the *t*-test. Non-normal distribution continuous data were presented as median (interquartile range) and tested by the Mann Whitney *U*-test. Significant *P* was set at < 0.05.

## Results

Fifty-one patients met the inclusion criteria and were enrolled, including 29 men and 22 women aged 36–71 years (mean 54.8 ± 8.3) (Table 1). Hypertension was present in 37 patients, diabetic mellitus in 22, atherosclerotic and coronary heart disease in 11; 15 had a past history of cerebral infarction. Forty-one of the patients were hospitalized because of cerebral infarction and two because of transient ischemic attacks. All the patients had digital subtraction angiography (DSA) before the stenting procedure. All the patients enrolled had severe stenosis at the MCA M1 segment, with the stenosis length ranging from 5.1 to 12.8 mm (mean 9 ± 3.3).

**Table 1:** Demography and stent treatment of the patients with MCA stenosis.

**Variables**		**Data**
Patients	Number	51
	F/M	22/29
	Age (y)	54.8 ± 8.3(36–71)
Prestenting	Stenosis location	M1 segment
stenosis	Stenosis degree	(87.5 ± 9.6)%
	Stenosis length	5.1–12.8 mm (mean 9.0 ± 3.3)
Reasons for	Cerebral infarction	18
hospitalization	Transient ischemic attack	2
Past history	Hypertension	37 (72.5%)
	Diabetic mellitus	22 (43.1%)
	Coronary heart disease	11 (21.6%)
	Cerebral infarction	15 (29.4%)
Predilatation	Predilatation balloon	Gateway
balloon	Balloon diameter (mm)	1.50–2.75 (mean 1.70 ± 0.20)
	Balloon length (mm)	9–20 (mean 13.5 ± 2.6)
	Dilatation pressure (atm)	2.0–8.0 (mean 5.3 ± 2.4)
	Dilatation time (s)	30–75 (mean 45.7 ± 34.8)
Poststenting	Poststent stenosis	31.4 ± 12.5%
	Residual stenosis	22.4 ± 3.5%
Complications	Perforator infarction	1 (1.96%)
Follow-up	Duration	3–12 (mean 5.8 ± 0.4)
	Instent stenosis	1 (1.96%)
	Ischemic event	1 (1.96%)

MCA, middle cerebral artery.

**Figure 2:**
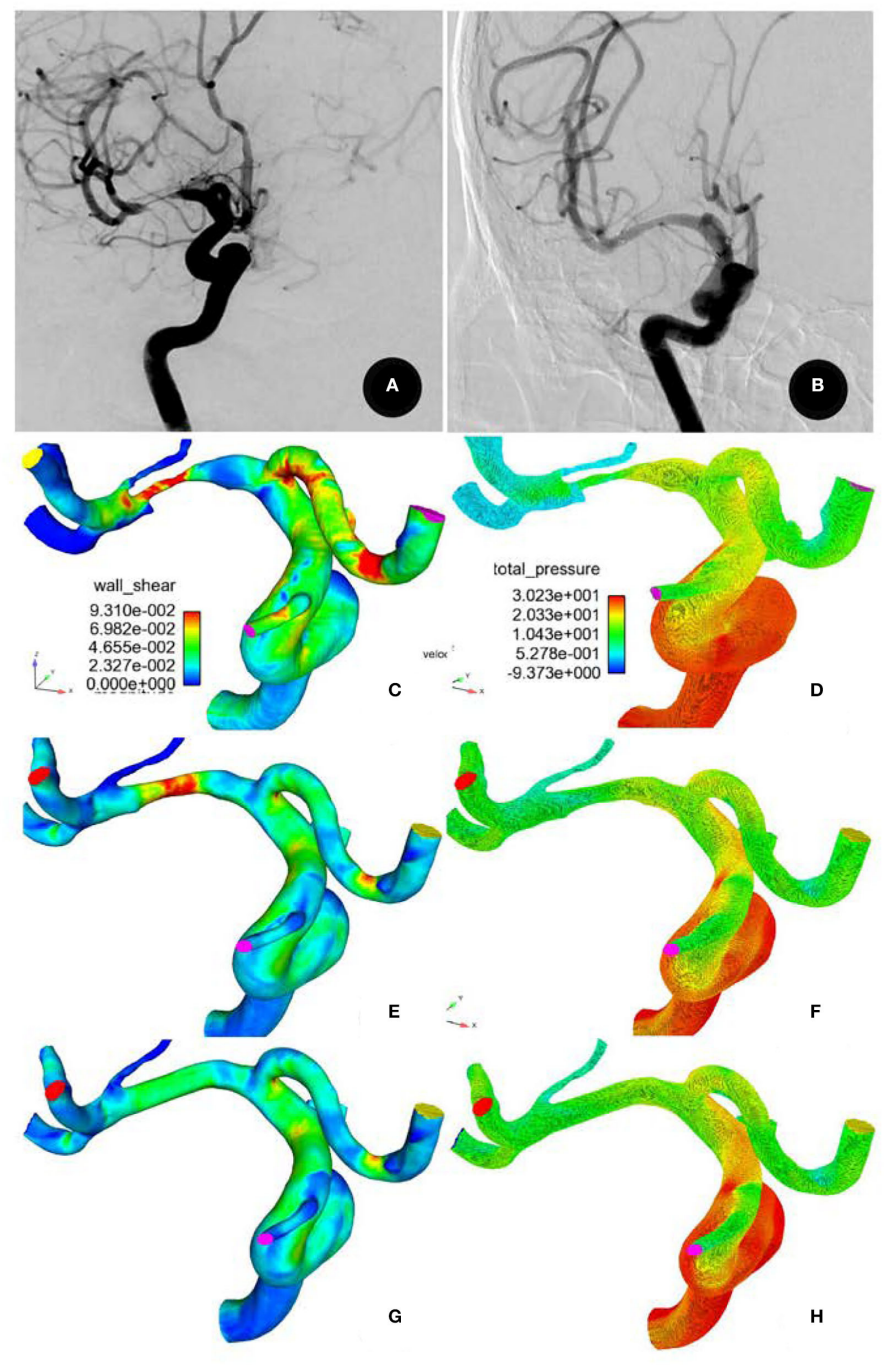
A 71-year-old male patient had severe stenosis at the M1 segment of the right middle cerebral artery. **(A)** Digital subtraction angiography showing the stenosis before stenting and **(B)** patent artery 6 months after recanalization **(B)**. **(C,D)** Before stenting, the total pressure was significantly higher, but the wall shear stress (WSS) was significantly decreased at the normal arterial segment proximal to the stenosis. At the stenosis and the perforator root, the total pressure was significantly decreased, but the WSS was significantly increased. Decreased WSS and pressure were present in all arteries distal to the stenosis. **(E,F)** After stent recanalization, the total pressure was significantly decreased, while the WSS proximal to the stenosis was increased. Both the total pressure and the WSS were significantly decreased at the stenosis but were increased at arteries distal to the stenosis. The total pressure was significantly increased, while the WSS was decreased at the perforator root. **(G,H)** After virtual repair, similar changes were observed in the total pressure and WSS.

Stent angioplasty was successful in all (100%) the patients (Figure 2). The angiography immediately after stenting demonstrated a significant (*P* < 0.05) decrease in MCA stenosis after comparison with before stenting (31.4 ± 12.5% vs. 87.5 ± 9.6%), with a residual stenosis of 15–30% (mean 22.4 ± 3.5%). Perforator infarction occurred in one (1.96%) patient after the procedure, but there were no deaths. At 3–12 month follow-up (mean 5.8 ± 0.4), one (1.96%) patient had an asymptomatic in-stent restenosis, and one (1.96%) patient presented with an ischemic event consistent with the blood supply area of the arterial stenosis.

A computational fluid dynamics analysis was performed before and after stenting and after virtual stenosis repair (Figure 2 and Table 2). In comparison with the hemodynamic parameters at the stenosis before stenting, the total pressure was significantly (*P* < 0.0001) elevated while the WSS, velocity, and vorticity were all significantly (*P* < 0.0001) decreased at the normal arterial segment proximal to the stenosis. The total pressure, WSS, velocity, and vorticity were all significantly (*P* < 0.0001) decreased at the normal arteries distal to the stenosis compared with those at the stenosis.

**Table 2:** Comparison of hemodynamic parameters before and after stenting (mean ± SD).

**Parameters**		**Pre-stenting**	**Post-stenting**	**Virtual repair**
Proximal to stenosis	WSS (Pa)	0.023 ± 0.009	0.042 ± 0.011*	0.046 ± 0.010*#
	Total pressure (Pa)	17.85 (17.1–19.9)	10.91 ± 2.38*	9.27 ± 2.21*
	Velocity (m/s)	0.06 (0.04–0.08)	0.10 (0.08–0.13)*	0.12 (0.10–0.14)*#
	Vorticity (1/S)	262.97 ± 104.61	386.6 ± 119.6*	417.2 ± 124.5*#
Stenosis	WSS (Pa)	0.120 ± 0.035	0.096 ± 0.014*	0.055 ± 0.005*#
	Total pressure (Pa)	12.84 ± 3.76	6.46 ± 1.21*	6.41 ± 0.83*
	Velocity (m/s)	0.20(0–0.24)	0.22(0.19–0.24)	0.16(0.15–0.17)*#
	Vorticity (1/S)	975.8 ± 303.4	688.5 ± 166.5*	504.6 ± 118.0*#
Perforator root	WSS (Pa)	0.012 (0.007–0.022)	0.001 (0.0008–0.002)*	0.006 (0.003–0.012)*#
	Total pressure (Pa)	0.09 (−0.66–1.98)	−0.55 (−0.57–−0.53)*	−0.24 (−0.65–0.66)*#
	Velocity (m/s)	0.023 (0–0.04)	0.012 (0–0.016)*	0.01 (0–0.03)*#
	Vorticity (1/S)	289.1 ± 118.5	77.6 ± 25.1*	168 (129–251)*#
Distal to stenosis	WSS (Pa)	0.027(0.002–0.059)	0.036 ± 0.010*	0.053 ± 0.006*#
	Total pressure (Pa)	0.45 (0.17–3.54)	3.77 (1.86–4.88)*	4.67 ± 1.55*#
	Velocity (m/s)	0.02 (0.01–0.11)	0.14 (0.11–0.15)*	0.16 (0.15–0.17)*#
	Vorticity (1/S)	220 (145.3–327)	384.1 ± 113.1*	437.7 ± 118.2*#

Data are presented as mean ± standard deviation or median (interquartile range). *P < 0.0001 compared with pre-stenting; #P < 0.0001 compared with post-stenting. Negative values of the perforator root indicate that the total pressure is in the opposite direction.

After the stent angioplasty, the WSS, total pressure, and vorticity at the stenosis location were all significantly (*P* < 0.0001) decreased, while the velocity was insignificantly higher (*P* > 0.05) than that before the stenting. At the normal arterial segment proximal to the stenosis, the WSS, velocity, and vorticity were all significantly (*P* < 0.0001) higher, while the total pressure was significantly (*P* < 0.0001) decreased as compared with that before the stenting. At the normal arterial segment distal to the stenosis, the WSS, total pressure, velocity, and vorticity were all significantly higher than those before the stenting. At the root of the perforating branch, the WSS, velocity, and vorticity were significantly decreased, while the total pressure was significantly higher (negatively) in the opposite direction than that before the stenting.

After the M1 stenosis was virtually repaired to normal arterial diameter, similar changes in the hemodynamic stresses were observed at all the locations compared with those after stent angioplasty. Compared with the optimal hemodynamic stresses after virtual stenosis removal, the hemodynamic stresses at all locations had recovered after the stenting even though some differences still existed.

## Discussion

In this study investigating the effect of endovascular recanalization by stent angioplasty on hemodynamic stresses for severe stenoses of the middle cerebral artery (MCA) M1 segment, it was found that stent angioplasty could significantly restore the atherosclerotic stentosis and that sub-satisfactory recanalization could significantly improve all the hemodynamic stresses proximal or distal to the stenosis and at the perforator root as compared with those before the stenting and were similar to those after virtual stenosis removal.

The blood supply region of the MCA is extensive, and the area of the MCA blood supply contains important functions of the brain. Arterial stenosis of the MCA often results in ischemic stroke, because perforating arterial branches distal to the stenosis usually lack blood compensation from collateral circulation and are more sensitive to blood flow changes. Endovascular recanalization by stent angioplasty can effectively improve and correct the hemodynamic disorder distal to the stenosis, and the blood flow of the distal perforating arterial branches can also be improved (23, 24).

Currently, three-dimensional imaging examinations, including magnetic resonance imaging angiography and DSA, can be performed to diagnose intracranial arterial atherosclerotic stenoses (25–27). With these imaging data, detailed information of intracranial arterial diseases can be obtained, and CFD analysis can also be performed using these three-dimensional imaging data. By CFD analysis, hemodynamic stresses, including total pressure, WSS, vorticity, and velocity at the stenosis and surrounding arterial segments can be determined before and after stent angioplasty and after virtual stenosis removal. By comparing the hemodynamic stresses at different time points, the effect of stent angioplasty on hemodynamic status can be investigated in detail.

Arterial stenosis will limit blood flow from going through the stenosis, and a great deal of blood will be congested proximal to the stenosis, leading to increase in pressure and decrease in WSS, flow speed, and vorticity proximal to the stenosis, as proved by the findings of our study. Because of higher pressure proximal to the stenosis, the speed of blood flow at the stenosis is significantly accelerated, resulting in increase in WSS and flow vorticity but decrease in total pressure. Distal to the stenosis, the arterial diameter returns to normal, and the WSS, total pressure, flow velocity, and vorticity will all be decreased because of reduced amount of blood flow from the stenotic upstream area. These hemodynamic changes have been proved at the M1 stenosis and normal segments proximal to the stenosis in our study.

After the sub-satisfactory recanalization of the stenosis by stent angioplasty, the stenotic arterial lumen is expanded to nearly normal. With increase in the amount of blood flow through the stented segment, the flow speed increases, the WSS and vorticity are enhanced, but the total pressure is decreased at the proximal segment. At arterial segments distal to the stented segment, all the hemodynamic stresses (WSS, total pressure, velocity, and vorticity) are increased because of higher amount of blood flow and flow speed. The increase in the total pressure and velocity of blood flow, thus, significantly promotes cerebral blood perfusion, improving the ischemic status of the brain and subsequent ischemic symptoms. This is the hemodynamic effect of stent angioplasty on intracranial atherosclerotic stenosis as confirmed in our study. At the root of the perforating arterial branch, the total pressure is negatively enhanced, and the vorticity is significantly decreased, which will promote blood flow into the perforating artery even though the flow speed is decreased. With improvement in the hemodynamic stresses and parameters after sub-satisfactory recanalization, the pre-stenting poor blood perfusion in the brain distal to the stenosis will be significantly improved, and the clinical prognosis will consequently be improved. On the follow-up of the patients 3–12 months (mean 5.8 ± 0.4) later, one (1.96%) had an asymptomatic in-stent restenosis, and one (1.96%) presented with an ischemic event consistent with the blood supply area of the arterial stenosis. These clinical outcomes may prove the efficacy of sub-satisfactory recanalization of severe MCA stenosis.

After the virtual removal of the arterial stenosis, the hemodynamic stresses are further improved, and even if the stresses have not completely recovered to the optimal status after the virtual removal, the sub-satisfactory stent recanalization has led to a significant improvement in the hemodynamic stresses, with improved cerebral perfusion pressure and blood flow speed.

Current hemodynamic studies are mainly focused on the primary technique of simulating the stenosis itself and WSS and pressure at the stenosis without revealing more hemodynamic stresses proximal and distal to the stenosis and at the root of a perforating branch (28–32), and hemodynamic stresses of arterial segments proximal and distal to the stenosis and at the perforating arterial branch are also worth studying, because they also play a very important role in the prognosis of these patients. As revealed by our study, the hemodynamic stresses proximal and distal to the stenosis and at the root of a perforating artery had been significantly improved after sub-satisfactory recanalization, thus promoting cerebral blood perfusion and improving the ischemic status of the brain tissue distal to the stenosis or supplied by the perforating artery. Thus, for patients who have M1 stenoses but no cerebral infarction in a large area, stent angioplasty of the stenosis will significantly improve cerebral hypoperfusion by ameliorating the hemodynamic status at and around the stenosis even though the hemodynamic stresses of sub-satisfactory recanalization have not been restored to the optimal status after virtual stenosis removal. From the perspective of hydrodynamics, this study has proved the clinical value of “sub-satisfactory” recanalization by stent angioplasty for patients with severe stenosis of the MCA M1 segment (13, 33–35).

Some limitations may exist in this study, including the limited number of patients, only Chinese patients were enrolled, being a single-center study, and stenosis only at the middle cerebral artery, which may all affect the generalization of the outcome. Future studies will have to resolve all these issues for better outcomes.

In conclusion, sub-satisfactory recanalization by stent angioplasty can significantly improve the hemodynamic status proximal and distal to the stenosis and at the perforator root as compared with that before stenting, similar to that after virtual stenosis removal, improving the hemodynamic status and cerebral hypoperfusion.

## Data availability statement

The original contributions presented in the study are included in the article/supplementary material, further inquiries can be directed to the corresponding author.

## Ethics statement

The studies involving human participants were reviewed and approved by Ethics Committee of Henan Provincial People's Hospital. The patients/participants provided their written informed consent to participate in this study. Written informed consent was obtained from the individual(s) for the publication of any potentially identifiable images or data included in this article.

## Author contributions

KZ and B-LG: study design. KZ, WR, J-CX, H-LG, Y-FW, and J-JG: data collection. KZ, Z-LW, and B-LG: data analysis. J-JG: supervision. KZ: writing of the original article. B-LG: revision. All authors contributed to the article and approved the submitted version.

## Funding

This study was funded by Joint Construction Project of Henan Provincial Medical Science and Technology Research Plan (SBGJ202003006).

## Conflict of interest

The authors declare that the research was conducted in the absence of any commercial or financial relationships that could be construed as a potential conflict of interest.

## Publisher's note

All claims expressed in this article are solely those of the authors and do not necessarily represent those of their affiliated organizations, or those of the publisher, the editors and the reviewers. Any product that may be evaluated in this article, or claim that may be made by its manufacturer, is not guaranteed or endorsed by the publisher.
